# Filetlappentransfer als Alternative zu konventionellen Amputationen der unteren Extremität

**DOI:** 10.1007/s00113-024-01460-y

**Published:** 2024-07-10

**Authors:** L. Harnoncourt, C. Gstoettner, L. Pflaum, G. Laengle, O. C. Aszmann

**Affiliations:** 1 Klinisches Labor für Bionische Extremitätenrekonstruktion, Universitätsklinik für Plastische, Ästhetische und Rekonstruktive Chirurgie, Medizinische Universität Wien, Wien, Österreich; 2 Universitätsklinik für Plastische, Ästhetische und Rekonstruktive Chirurgie, Medizinische Universität Wien, Währinger Gürtel 18–20, 1090 Wien, Österreich

**Keywords:** Untere Extremität, Amputationsstümpfe, Körperbild, Nervenschmerzen, Sensibilität, Lower limb, Amputation stumps, Body image, Neuralgia, Sensation

## Abstract

**Hintergrund:**

Die prothetische Versorgung nach einer Amputation der unteren Extremität geht mit einigen Herausforderungen einher. Skeletale Stumpf-Prothesen-Schnittstellen und selektive Nerventransfers können diese teilweise bewältigen, bringen jedoch auch Einschränkungen, die die Notwendigkeit neuer Ansätze unterstreichen, mit sich. Hier kann das Konzept der sog. Ersatzteilchirurgie mit der Nutzung von Filetlappen eine wichtige Rolle spielen.

**Fragestellung:**

Übersicht über die klassischen prothesenassoziierten Beschwerden, Vor- und Nachteile von Versorgungsstrategien sowie Präsentation alternativer chirurgischer Konzepte.

**Material und Methoden:**

Es erfolgte eine selektive Literaturrecherche unter Berücksichtigung eigener Erfahrungen und Ansichten bezüglich Vor- und Nachteilen der chirurgischen Versorgungsmöglichkeiten. Zusätzlich wird ein klinischer Patientenfall vorgestellt.

**Ergebnisse und Schlussfolgerung:**

Der Transfer der Fußsohle als Filetlappen in die Belastungszone des Amputationsstumpfes geht mit einer Vielzahl von Vorzügen wie Endbelastbarkeit des Stumpfes, Vorbeugen von Nervenschmerzen, erhaltener Sensibilität und Bewahren des Körperbilds einher. Die Technik kann bei Amputationen sowohl proximal wie auch distal des Kniegelenks eingesetzt werden, vorausgesetzt, dass die Fersenregion nicht beeinträchtigt ist. Die Frage, ob Anteile des Knochens in den Transfer einbezogen werden soll, ist bei jedem Patienten individuell zu evaluieren. Dieser Ansatz ermöglicht die Optimierung des Amputationsstumpfes für die nachfolgende prothetische Versorgung der Patienten.

## Abstract (en)

**Background:**

The fitting of a prosthesis after lower limb amputation is associated with several challenges. Skeletal stump-prosthesis interfaces and selective nerve transfer can partially overcome these but are also associated with new impairments that emphasize the necessity of innovative approaches. The concept of so-called spare part surgery with the use of fillet flaps could play an important role in this respect.

**Objective:**

An overview of the classical prosthesis-associated discomforts, advantages and disadvantages of treatment strategies and presentation of alternative surgical concepts.

**Material and methods:**

A selective literature search was carried out considering the experiences of the authors and perspectives with respect to the advantages and disadvantages of the surgical treatment options. Furthermore, a clinical case is presented.

**Results and conclusion:**

The transfer of the sole of the foot as a fillet flap to the weight-bearing region of the amputation stump offers a number of benefits, such as creating a fully weight-bearing stump, prevention of neuralgia, preserved sensation and conservation of the body image. As long as the calcaneal region is not impaired, this technique can be performed in amputations below as well as above the knee. The question of whether parts of the bone should be included in the transfer must be individually evaluated for each patient. This approach enables optimization of the residual limb stump for the subsequent fitting of a prosthesis for the patient.

Amputationen stellen für Patienten einschneidende Erlebnisse mit schwerwiegenden Auswirkungen auf die weitere Lebensgestaltung dar. Eine zuverlässige und komplikationsarme prothetische Versorgung ist daher besonders wichtig. Klassische Schaftprothesen gehen jedoch oftmals mit einer Vielzahl von Komplikationen einher. Für eine regelmäßige Prothesennutzung ist es von Bedeutung, diese Beschwerden im Stumpfbereich an der Schnittstelle zwischen Mensch und Prothese zu minimieren, um den Betroffenen die bestmögliche Reintegration in den Alltag zu ermöglichen.

## Hintergrund

Der Verlust der unteren Extremität bedeutet für Patienten eine erhebliche Beeinträchtigung ihrer Lebensqualität, hat grundlegende Auswirkungen auf ihre Alltagsgestaltung und geht mit ausgeprägten psychosozialen Folgen einher [3]. Die Anpassung von Prothesen für Amputationen oberhalb des Sprunggelenks stellt oftmals eine Herausforderung dar, insbesondere im Hinblick auf eine langfristig zufriedenstellende Lösung. Der Weichteilmantel im Bereich des Amputationsstumpfes muss hohen Belastungen standhalten, ist jedoch im Vergleich zur dicken und widerstandsfähigen Haut der Fußsohle relativ dünn und nicht darauf ausgelegt, diesen Kräften gewachsen zu sein. Zusätzlich ist die Belastungszone auf eine geringe Fläche reduziert und die Druckbelastung beträchtlich erhöht. Infolgedessen kann es zu chronischen Weichteilschäden am Stumpf sowie zu einer unzuverlässigen Kraftübertragung auf die Prothese kommen.

Für die optimale Versorgung der Prothese-Bein-Schnittstelle gilt: einen sensiblen und endbelastbaren Stumpf zu gewährleisten

Prothesenschäfte werden daher häufig an proximalen Knochenvorsprüngen abgestützt, was wiederum mit Schmerzen und zu Einschränkungen der Bewegungsfreiheit verbunden sein kann. Darüber hinaus können sich im distalen Stumpfbereich druckempfindliche und schmerzhafte Neurome bilden. Insgesamt können diese Komplikationen zu einer reduzierten und selteneren Nutzung und in manchen Fällen zur vollständigen Ablehnung der Prothese führen [16]. Chirurgische Konzepte, wie Targeted Muscle Reinnervation (TMR) zur Neuromprophylaxe oder Osseointegration (OI) zur skeletalen Verankerung der Prothese, können diese Komplikationen erfolgreich reduzieren, bringen jedoch auch Nachteile mit sich. Zur optimalen Versorgung der Schnittstelle zwischen Prothese und Bein bzw. der Belastungszone lässt sich der Grundsatz der plastischen Chirurgie „Gleiches mit Gleichem ersetzen“ verfolgen.

## Herausforderungen nach Amputation und klassischer Prothesenversorgung

**Abb. 1:**
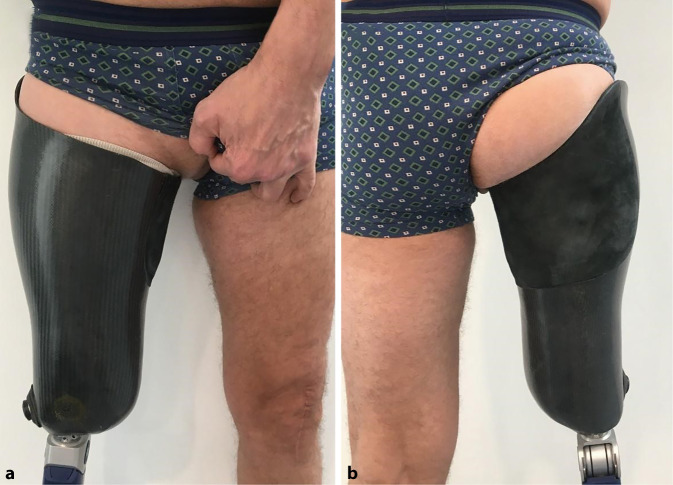
Bei konventionellen Schaftversorgungen nach transfemoraler Amputation ist neben einer weiten Einfassung des Stumpfes eine zusätzliche Abstützung am Beckenknochen notwendig. Ansichten von ventral (**a**) und dorsal (**b**)

Bei herkömmlichen Prothesenversorgungen der unteren Extremitäten wirken hohe Kräfte auf die Haut und den Weichteilmantel des Stumpfes, wodurch eine weite Einfassung der Restextremität notwendig ist. Die klassischen Begleitbeschwerden solcher Schaftversorgungen sind Schmerzen, übermäßiges Schwitzen und Hautirritationen [11] sowie Bewegungseinschränkungen, wenn das Kniegelenk in die Versorgung einbezogen werden muss. Hierbei muss je nach Patient und Amputationshöhe individuell zwischen Bewegungsfreiheit und Belastung am Stumpf abgewogen werden. Transtibiale Amputationen ermöglichen den Erhalt des Kniegelenks und eine größere Bewegungsfreiheit bei geringerer Gewichtsbelastung. Um bei der Versorgung transfemoraler Amputationen einen sicheren Sitz zu gewährleisten sowie Druckstellen und -defekte zu vermeiden, muss das Gewicht auf belastbare Bereiche umverteilt werden, da der Stumpf allein nicht endbelastbar ist und der Knochenstumpf zu Läsionen der Haut führen würde. Aufgrund besserer Stabilität und geringerer Fallneigung hat sich das ischial gelagerte Design, bei dem der Sitzbeinhöcker als Gegenlager zum medialen Rand des Schafts dient, durchgesetzt (Abb. 1). Die Größe des Schafts und der erforderliche Kontakt zum Beckenknochen beschränken jedoch den Tragekomfort und die Bewegungsfreiheit, stören das Gangbild und führen häufig zu Schmerzen im Bereich der Beckenabstützung, einschließlich Hautirritationen der dort überbelasteten Haut [7].

**Abb. 2:**
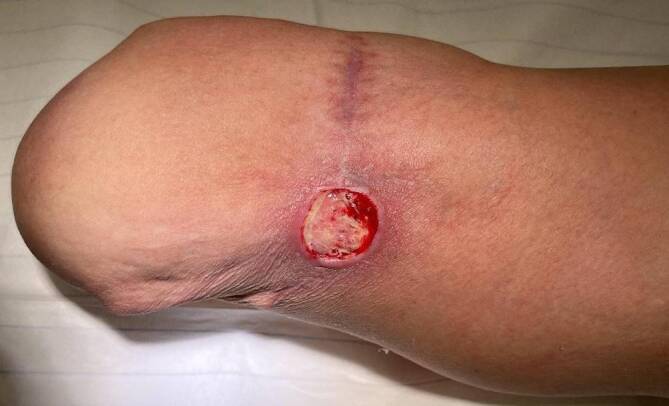
Die chronische Belastung kann im Stumpfbereich zu schmerzhaften Weichteildefekten und Ulzerationen führen

Stumpfschmerzen treten bei etwa 60 % der Patienten auf und werden nach somatischer und neuropathischer Ätiologie unterschieden [22]. Somatische Stumpfschmerzen entstehen durch Druckbelastung, chronische Ulzerationen, knöcherne Auswüchse und vaskuläre Insuffizienz (Abb. 2). Neuropathische Schmerzen werden durch Neurome ausgelöst [22]. Nach Durchtrennung der Nerven im Rahmen der Amputation regenerieren dessen Axone richtungslos, da das distale Nervenziel fehlt, sodass es zu einer knollenförmigen Hyperplasie von Nervengewebe, einem Neurom, kommt [13, 18]. Die Folgen persistierender Stumpfschmerzen sind verringerte Mobilität, reduzierte körperliche Aktivität, geringere Teilnahme an Aktivitäten des täglichen Lebens sowie daraus resultierende psychische und physische Auswirkungen [20].

Bei vielen Betroffenen treten zusätzlich Phantomschmerzen, projizierte Schmerzempfindungen der amputierten Gliedmaße, auf [15]. Es konnte nachgewiesen werden, dass das regelmäßige Tragen einer Prothese die Schmerzen signifikant reduziert [2] und zudem die Wiederherstellung von sensorischem Feedback am Stumpf Phantomschmerzen verringern sowie die Funktionalität verbessern kann [10].

## Chirurgische Versorgungskonzepte

Um die beschriebenen Begleitbeschwerden der prothetischen Versorgung nach Amputationen der unteren Extremität ober- bzw. unterhalb des Knies zu bewältigen, wurden im Lauf der letzten Jahre neben orthopädietechnischer Innovation auch chirurgische Lösungsansätze präsentiert.

### Osseointegration

**Abb. 3:**
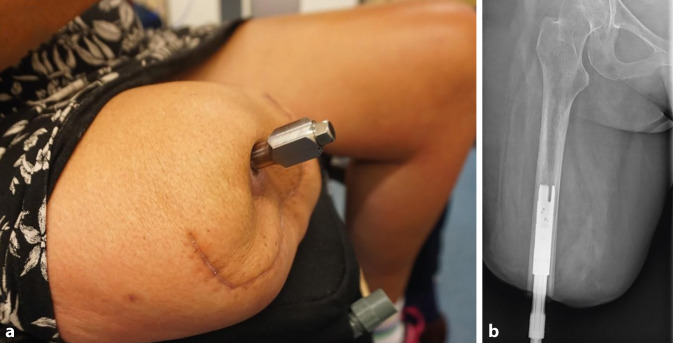
**a** Amputationsstumpf nach durchgeführter Osseointegration; **b** das Röntgenbild zeigt die Fixierung des Implantats im Femur

Das Konzept der Osseointegration (OI), der skeletalen Anbindung der Prothese, bietet eine Möglichkeit, die klassische Schaftversorgung als Schnittstelle zwischen Stumpf und Prothese zu ersetzen. Eine weite Einfassung des Stumpfes mit allen verbundenen, angeführten Nachteilen wird vermieden und gleichzeitig der Weichteilmantel entlastet. Diesbezüglich existieren verschiedene Systeme auf dem Markt. Eine Technik beruht auf einem Titanimplantat („fixture“), das in den Knochen am Stumpf eingebracht wird. Nach entsprechender Einheilung und Rehabilitation kann die Prothese über ein perkutanes Verbindungsstück („abutment“) befestigt werden und ist direkt im Knochen verankert (Abb. 3; [5]).

Insbesondere nach transfemoralen Amputationen findet die OI seit vielen Jahren Anwendung, und es ist inzwischen eine Vielzahl positiver Effekte beschrieben [6]. Verglichen mit Schaftprothesen, führt die OI zu längerer Tragedauer, höherem Komfort, verbessertem Bewegungsausmaß und erhöhter Lebensqualität [19]. Ein weiterer wesentlicher Vorteil der OI besteht darin, dass das Gelenk bei Patienten mit sehr kurzem Stumpf nach gelenknahem Extremitätenverlust auch funktionell erhalten werden kann. Eine konventionelle Schaftversorgung würde hingegen zum Verlust der bestehenden Gelenkfunktion führen.

Trotz beträchtlicher Vorteile der OI gibt es doch auch limitierende Faktoren zu beachten

Trotz dieser ersichtlichen Verbesserungen der prothetischen Versorgung nach OI stehen einer breiteren Implementierung in den klinischen Alltag noch diverse limitierende Faktoren im Weg. Hohe Kosten und die notwendige Expertise für diese anspruchsvolle Technik ermöglichen den Einsatz aktuell nur in wenigen, spezialisierten klinischen Zentren. Zusätzlich besteht aufgrund des perkutanen Durchtritts ein erhöhtes Risiko für Infektionen, die eine medikamentöse Therapie bis hin zur chirurgischen Revision oder sogar einer Entfernung des Implantats zur Folge haben können [6]. Darüber hinaus ist insbesondere das Abutment anfällig für mechanische Schäden, beispielsweise beim Stolpern oder Stürzen [6]. Neben dem Risiko einer Schädigung des Implantatsystems ist eine intensive und sportliche Belastung der Prothese bei OI gemäß der Erfahrung der Autoren nicht immer möglich. Aufgrund der direkten Lastübertragung auf den harten Knochen sind sportliche Aktivitäten mit intensivem Einsatz der unteren Extremität, beispielsweise Joggen, für einige Patienten schlichtweg zu schmerzhaft; eine Einschränkung, die bei optimaler konventioneller prothetischer Versorgung nicht besteht [4].

### Targeted Muscle Reinnervation

Die Targeted Muscle Reinnervation (TMR) wurde 2004 erstmals von Kuiken et al. in der oberen Extremität vorgestellt und beschreibt die chirurgische Umlagerung von Nerven, die nach einer Amputation ihre Zielorgane verloren haben, auf neue Muskeln. Primäres Ziel dieser Intervention war das Schaffen neuer Muskelsignale im Stumpfbereich zur verbesserten Steuerung myoelektrischer Prothesen der oberen Extremität [21]. Darüber hinaus werden mithilfe der durchgeführten Nerventransfers auch Nervenschmerzen effektiv behandelt. Bestehende Neurome werden reseziert und die Nerven auf die motorischen Äste der entsprechenden Muskeln verlagert. Die Nerven erhalten ein neues Zielorgan, wodurch einer neuerlichen Neurombildung vorgebeugt [12, 17, 25] und von den Patienten zusätzlich eine Besserung der bestehenden Phantomschmerzen angegeben wird [12, 17].

Keinen Zielnerven findende Axone können erneute Neurombeschwerden auslösen

In der Indikation zur Schmerztherapie findet die TMR auch Anwendung im Bereich der unteren Extremität [13]. Die von manchen Autoren vorgeschlagene [27] standardmäßig präventive Anwendung von TMR im Rahmen der Amputation der unteren Extremität ist fraglich, da nicht alle Patienten Phantom- bzw. Neuromschmerzen entwickeln [8]. Der Eingriff erfordert zudem ein hohes Maß an Expertise und verlängert die Operationszeit, wodurch das Risiko für mögliche Komplikationen, wie beispielsweise Infektionen, oder auch die Kosten steigen. Darüber hinaus besteht beim Transfer der großen Nervenstümpfe auf die kleineren motorischen Äste meist ein erheblicher Größenunterschied. Es erscheint daher unwahrscheinlich, dass sämtliche Nervenfasern vollständig in den kleineren Zielnerven einwachsen und ihnen tatsächlich ein neues Ziel angeboten wird. Stattdessen wird vermutet, dass eine Vielzahl der regenerierenden Axone in benachbarte Bereiche, beispielsweise direkt in die angrenzende Muskulatur, eindringt bzw. kein Ziel findet und zur erneuten Bildung von Neurombeschwerden führen kann [14].

#### Fazit.

- Die vorgestellten Konzepte konnten durch Auflösung vieler klassischer schaftvermittelter bzw. Postamputationsbeschwerden bereits eine deutliche Verbesserung der prothetischen Versorgung von Amputationen der unteren Extremität erzielen.
- Sie bringen jedoch auch neue Herausforderungen mit sich und lassen manche Komplikationen ungelöst, wodurch die Notwendigkeit anderer Ansätze ersichtlich wird.

## Altbekannte Konzepte als innovative Lösungsansätze

### „Spare-Part“-Chirurgie

Eine Möglichkeit der Stumpfversorgung und -optimierung zur anschließenden prothetischen Versorgung ist die „Spare-Part“- oder „Ersatzteil“-Chirurgie. Gewebe des ansonsten verworfenen Amputats wird als sog. Filetlappen wiederverwendet, entweder gefäßgestielt oder als freier mikrovaskulärer Gewebetransfer. Das Konzept wurde erstmals von Moberg in der unteren Extremität zur Deckung einer chronischen Läsion an der Ferse mit einem Hallux-Filet-Lappen beschrieben [24] und findet seither sowohl bei traumatologischen [23] als auch onkologischen [1] Indikationen Anwendung. Filetlappen können sowohl an der oberen als auch an der unteren Extremität eingesetzt werden; die Lappengröße reicht von kleinen digitalen Lappen bis hin zu sehr ausgedehnten Lappen, die große Teile des Weichteilmantels einer Extremität umfassen. Obwohl diese Möglichkeit im Rahmen einer Amputation oftmals nicht bedacht wird, kann ihre Anwendung zur Deckung des proximalen Stumpfes ohne zusätzliche Hebemorbidität genutzt werden.

**Abb. 4:**
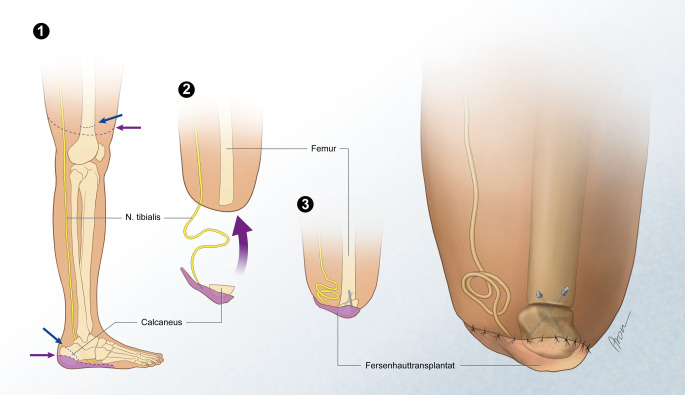
Schematische Darstellung eines nervengestielten Fersenhauttransfers mit Anteilen des Calcaneus

Dieses Konzept wurde an der Universitätsklinik für Plastische, Ästhetische und Rekonstruktive Chirurgie der Medizinischen Universität Wien aufgegriffen und bei Patienten mit subakuten oder elektiven Ober- oder Unterschenkelamputationen mit unversehrter distaler Extremität verfolgt. Nach durchgeführter Amputation erfolgt, je nach Ausgangssituation, ein freier oder gestielter Gewebetransfer der Fersenhaut mit oder ohne Anteile des darunterliegenden Calcaneus an den distalen Amputationsstumpf (Abb. 4). Diese Intervention bietet zahlreiche Vorteile bei gleichzeitig geringem Risiko, da bei evtl. Lappennekrose auf eine konventionelle Stumpfbildung zurückgegriffen werden kann.

Durch den Transfer der Fersenhaut auf den Stumpf wird Gewebe, das physiologisch darauf ausgelegt ist, diesen Belastungen standzuhalten, in die Belastungszone verlagert. Es gelingt, einen robusten und endbelastbaren Stumpf zu schaffen. Diese Ausgangssituation bietet mehr Möglichkeiten im Hinblick auf die prothetische Versorgung, da es einer geringer dimensionierten Einfassung der Restextremität bedarf. Klassische schaftassoziierte Beschwerden können reduziert und gleichzeitig eine höhere Bewegungsfreiheit erzielt werden, da die proximale Gelenkebene nicht in die Versorgung einbezogen werden muss.

Ziele sind die Wiederherstellung der Endbelastbarkeit des Amputationstumpfes um damit der Erhalt der proximalen Gelenkkette

Gerade bei gelenknahen Amputationen kann darüber hinaus eine Defektdeckung und bei Mitnahme des Calcaneus auch eine Verlängerung des Restknochens unter Vermeidung einer zusätzlichen Hebemorbidität erzielt werden. Ziel ist es, das Gelenk funktionell zu erhalten und zusätzlich die Belastungszone durch Augmentation des Knochendurchmessers am Stumpf zu vergrößern.

Durch Koaptation der Stumpfnerven nach freiem Gewebetransfer oder Umlagerung eines neurovaskulär gestielten Lappens wird eine haptische Resensiblisierung im distalen Stumpfbereich erreicht. Die Nerven erhalten ein neues Ziel bzw. bleiben intakt, wodurch sowohl Neurombeschwerden als auch Phantomschmerzen vorgebeugt wird. Das sensible Feedback am distalen Stumpfende wird vom Patienten kognitiv im Bereich der Fußsohle wahrgenommen. Das Ende des Stumpfes bleibt sensibel folglich das Ende des Beins; dies erleichtert es dem Patienten, sein Körperbild und seine körperliche Integrität nach der Amputation zu bewahren bzw. wiederzuerlangen, was sich insbesondere auf Phantomschmerzen positiv auswirkt.

### Ergebnisse in der Literatur

Vereinzelte Berichte über derartige Lappenplastiken der Fußsohlenhaut nach transtibialen Amputationen finden sich in der Literatur [9, 26]. Das primäre Ziel der Intervention war die Deckung eines Defekts am Stumpf unter Vermeidung zusätzlicher Hebemorbidität. Gleichzeitig konnte das Gelenk erhalten und der Stumpf durch den Transfer von endbelastbarem, sensiblem Gewebe für die spätere prothetische Versorgung optimiert werden. Dies äußerte sich durch geringeres Auftreten klassischer prothesenassoziierter Komplikationen wie Schmerzen, Ulzerationen und Neuromen [26].

Die „Ersatzteil“-Chirurgie optimiert den Amputationsstumpf für die spätere prothetische Versorgung

Die Frage, ob das Fersenbein in den Transfer einbezogen werden soll, wird kontrovers diskutiert. Als Vorteil eines fasziokutanen Lappens wird eine kürzere Zeitspanne (etwa 40 Tage) bis zum Beginn des Rehabilitationsprozesses mit Belastung des Stumpfes genannt, und es wird auf das Risiko einer verzögerten oder unvollständigen Knochenheilung hingewiesen [9]. Verglichen damit war die Zeit bis zur Rehabilitation nach osteokutanem Transfer deutlich länger (80 bis 180 Tage, [26]). Außerdem verheilte der Knochen bei einem von 7 Patienten initial nicht, was jedoch mit einer Revisionsoperation behandelt werden konnte. Den Knochen einzubeziehen, soll insbesondere bei Amputationen mit kurzem Knochenstumpf erwogen werden, um die Länge der Restgliedmaße und die Gelenkfunktion zu verbessern [26]. Die diesbezügliche Indikation muss im Einzelfall sorgfältig geprüft werden.

### Klinisches Fallbeispiel

Der chirurgische Eingriff sowie erzielbare Ergebnisse sollen nachfolgend anhand eines Patientenbeispiels veranschaulicht werden. Bei dem Patienten handelt es sich um einen 49-jährigen Mann mit massiven rechtsseitigen Knieschmerzen, die jeglichen Einsatz des Gelenks und der gesamten unteren Extremität unmöglich machten. Im Alter von 5 Jahren hatte er bei einem Verkehrsunfall schwere Verletzungen des rechten Knies, die zu einer posttraumatischen Arthrose und in weiterer Folge zur Implantation einer Knieprothese führten, erlitten. Aufgrund auftretender Komplikationen hatte er ausgeprägte Knieschmerzen, die mehrere Revisionen und Implantatwechsel erforderlich machten.

Die Eingriffe blieben jedoch ohne Erfolg, und der Patient stellte sich in der Klinik mit Schmerzen in einer Intensität von 10 auf der Numeric Rating Scale (NRS) bei jeglicher, auch nur minimaler Belastung des betroffenen Beins vor. Im Rahmen der Vorstellung äußerte er den Wunsch nach einer elektiven Amputation oberhalb des Knies.

**Abb. 5:**
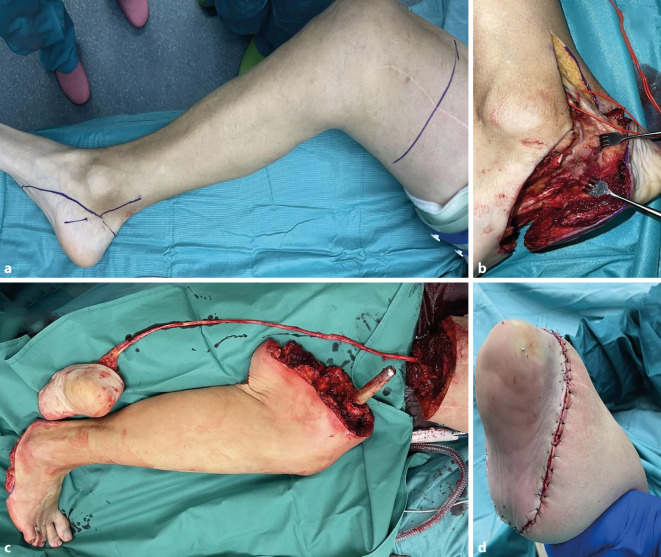
**a** Präoperative Markierung der Oberschenkelamputation mit Filetlappentransfer. **b** Präparation des Lappens, einschließlich Osteotomie des Calcaneus. **c** Nach der Amputation bleibt der N. tibialis zur Innervation der Ferse in Kontinuität. **d** Endresultat nach mikrovaskulärem Transfer des Filetlappens zum Oberschenkelstumpf

Da die Sensibilität der Fußsohle nicht beeinträchtigt war, wurde nach der Amputation ein neurogen gestielter Filetlappen der Fersenhaut, einschließlich des Fersenbeins, auf den Oberschenkel transferiert (Abb. 5). Die Osteosynthese (2 Kompressionsschrauben) musste aufgrund eines Sturzes mit resultierender Fraktur des Knochentransplantats revidiert werden, heilte danach jedoch schnell und komplikationslos aus. Die Belastung des Stumpfes war 6 Wochen nach der Revisionsoperation möglich.

**Abb. 6:**
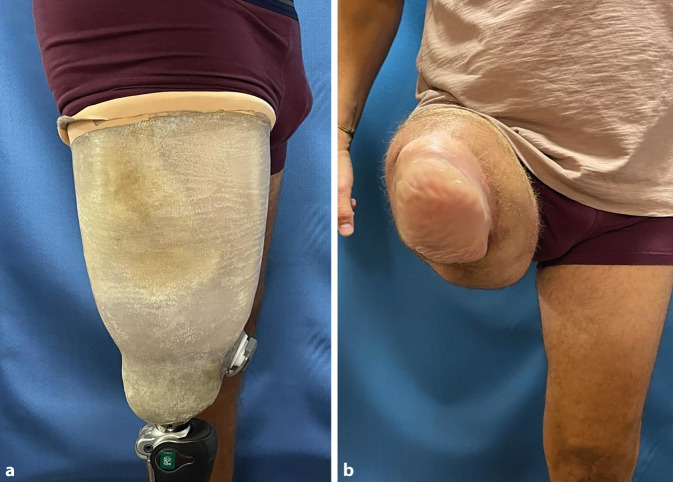
Der Patient bei einer Kontrolle etwa 5 Monate postoperativ. **a** Bereits die erste Probeversorgung ist geringer dimensioniert und kommt ohne Abstützung am Becken aus. **b** Der Lappen ist gut eingeheilt und vollständig sensibel

Bei einer Kontrolle etwa 5 Monate postoperativ war der Patient bereits mit einer provisorischen Prothese versorgt (Abb. 6). Die klinische Untersuchung ergab sowohl eine subjektiv als auch objektiv erhaltene Sensibilität der Fersenhaut am Stumpf. Durch den stabilen, endbelastbaren und sensiblen Stumpf kann der Patient trotz transfemoraler Amputation mit einer Prothese ohne Abstützung am Beckenknochen schmerzfrei, aber mit erhaltener Sensibilität gehen.

## Resümee

Der Verlust einer Extremität bedeutet für die Betroffenen ein einschneidendes Erlebnis, das neben sozialen und beruflichen Einschränkungen oftmals auch eine veränderte Selbstwahrnehmung und psychische Belastungen zur Folge hat [3]. Amputationen der unteren Extremitäten und ihre Versorgung stellen sowohl Patienten also auch behandelnde Ärzte und Orthopädietechniker vor Herausforderungen. Durch den Einsatz neuer Versorgungskonzepte wie OI und TMR können einige davon bewältigt werden, andere bleiben jedoch bestehen oder werden nur unzureichend adressiert. Der Transfer der intakten Fußsohlenregion als Filetlappen im Rahmen der Amputation der unteren Extremität bietet eine innovative Alternative und eine physiologische Rekonstruktion der Belastungszone zwischen Stumpf und Prothese, insbesondere im Hinblick auf Sensibilität, Schmerzmanagement und Endbelastbarkeit. Vorausgesetzt die Fersenregion ist unbeeinträchtigt, sollte dieses Konzept zur bestmöglichen Stumpfversorgung und optimalen Vorbereitung auf eine prothetische Versorgung nach Amputation der unteren Extremität angedacht werden.

## Fazit für die Praxis

- Klassische Prothesenversorgungen nach Amputationen der unteren Extremität gehen oftmals mit Beschwerden am Stumpf einher.
- Chirurgische Ansätze wie Osseointegration und Targeted Muscle Reinnervation können diese Beschwerden z. T. reduzieren, sind aber auch mit neuen Herausforderungen verbunden.
- Bei dem Konzept der Ersatzteil- oder „Spare-Part“-Chirurgie wird anderenfalls verworfenes Gewebe des Amputats als Filetlappen zur Defektdeckung oder zum Gewebeaufbau am Stumpf genutzt.
- Die Fußsohlenregion als Filetlappen kann zur Reduktion der somatischen und neuropathischen Stumpfschmerzen und zur Optimierung des Stumpfes im Hinblick auf Sensibilität und Belastbarkeit für die anschließende prothetische Versorgung dienen.
